# 

**Published:** 1898-06

**Authors:** 


### CONTENTS.

**ORIGINAL COMMUNICATIONS—**

MOUTH-BREATHING IN CHILDREN, PARTICULARLY AS A RESULT OF ADENOIDS. By Arthur G. Hobbs, M.D., Atlanta, Ga. .......... 217

SOME REMARKS UPON SYPHILITIC MANIFESTATIONS IN THE LARYNX. By Paul Turner Vaughan, M.D., Hot Springs, Ark .......... 223

PHTHISIS, OR PULMONARY TUBERCULOSIS. By J. L. Campbell, M.D., Atlanta, Ga.... 228

TWO CASES FROM MY NOTE-BOOK. By W. C. Galloway, M.D., Wilmington, N. C...... 235

**SOCIETY REPORTS—**

ATLANTA SOCIETY OF MEDICINE .......... 238

**CORRESPONDENCE—**

SPINAL CURVATURE AND GYMNASTICS .......... 42

**EDITORIAL—**

CONSERVATIVE GYNECOLOGY .......... 245

THE DEADLY(?) CIGARETTE .......... 247

SOUTHERN ASSOCIATION OF RAILWAY SURGEONS .......... 249

CONSOLIDATION OF THE ATLANTA MEDICAL COLLEGE AND THE SOUTHERN MEDICAL COLLEGE .......... 251

**MEDICAL ITEMS** .......... 252

**BOOK REVIEWS** .......... 258

**SELECTIONS AND ABSTRACTS** .......... 260

**MEDICAL ITEMS—CONTINUED** .......... x, xi, xii, xiii, xiv, xvi, xvi

**ADVERTISERS' INDEX TO ADVERTISEMENTS** .......... xviii

**CLASSIFIED INDEX TO ADVERTISEMENTS** .......... xix

